# Segmented Estimation of Road Adhesion Coefficient Based on Multimodal Vehicle Dynamics Fusion in a Large Steering Angle Range

**DOI:** 10.3390/s25072234

**Published:** 2025-04-02

**Authors:** Haobin Jiang, Tonghui Shen, Bin Tang, Kun Yang

**Affiliations:** School of Automotive Engineering Research Institute, Jiangsu University, Zhenjiang 212013, China; 2222204026@stmail.ujs.edu.cn (T.S.); tangbin@ujs.edu.cn (B.T.); yc8325@163.com (K.Y.)

**Keywords:** steer-by-wire system, road surface friction coefficient estimation, Adaptive Unscented Kalman Filter (AUKF) algorithm, extended state observer

## Abstract

Real-time estimation of the road surface friction coefficient is crucial for vehicle dynamics control. Under large steering angles, the accuracy of existing road surface friction coefficient estimation methods is unsatisfactory due to the nonlinear characteristics of the tire. This paper proposes a segmented estimation method for the road adhesion coefficient, which considers different steering angle ranges and utilizes multimodal vehicle dynamics fusion. The method is designed to accurately estimate the road adhesion coefficient across the full steering angle range of the steer-by-wire system. When the front wheel angle is small (less than 2.8°), an improved Unscented Kalman Filter (AUKF) algorithm is used to estimate the road surface friction coefficient. When the front wheel angle is large (greater than 3.2°), a rack force expansion state observer is constructed using the dynamics model of the steer-by-wire actuator to estimate the rack force. Based on the principle that the rack force varies with different road surface friction coefficients for the same steering angle, the rack force is used to distinguish the road surface friction coefficient. When the front wheel angle is between the two ranges, the average value of both methods is taken as the final estimate. The method is verified through Matlab/Simulink and CarSim co-simulation, as well as hardware-in-the-loop experiments of the steer-by-wire system. Simulation results show that the relative error of road surface friction coefficient estimation is less than 10% under different steering angles. The segmented combination estimation strategy proposed in this paper reduces the impact of tire nonlinearities on the estimation result and achieves high-precision road surface friction coefficient estimation over the entire steering angle range of the steer-by-wire system, which is of significant importance for vehicle dynamics control.

## 1. Introduction

With the rapid iteration and innovative development of automotive chassis technology, active safety technology in vehicles has gained increasing attention. The road surface friction coefficient, as an important indicator of the interaction between the tire and the road surface, forms the foundation for achieving precise control of vehicle motion. The steer-by-wire system is the mainstream development direction in automotive steering technology. It offers advantages such as variable steering angle ratio control, active front-wheel steering, meeting human–machine co-driving requirements, and supporting the development of advanced autonomous driving. For steer-by-wire systems, the real-time and precise estimation of the road surface friction coefficient over a large front wheel angle range has a direct and significant impact on vehicle trajectory tracking control, driving stability, and safety.

Scholars both domestically and internationally have conducted extensive research on estimating the road surface friction coefficient. Currently, the mainstream methods can be classified into two types: one involves measuring parameters related to the road surface friction coefficient using onboard sensors to determine it, while the other calculates the friction coefficient based on the relationship between vehicle dynamics and the coefficient. Research has shown that the road surface friction coefficient is influenced by both the micro and macro textures of the road surface [1]. To obtain this coefficient using this theory, a certain amount of road information must first be captured through cameras, followed by using algorithms to calculate the friction coefficient. Langstrand et al. [2] trained a neural network using a dataset of 20,000 RGB images and employed visual light sensors to detect road surfaces in real time for the precise estimation of the road surface friction coefficient. Otoofi et al. [3] used an end-to-end approach to learn a shared latent space that includes semantic segmentation and friction coefficient information, employing *geco to train the model for minimization.

This method effectively identifies friction coefficients of surfaces such as snow, ice, gravel, and dry asphalt. Yan et al. [4] proposed a real-time road surface friction coefficient (RSFC) detection method that combines the diffusion model and the TNT model. By enhancing the dataset, the accuracy of recognition was improved with a processing speed of 2 milliseconds. Experimental results show that this method improved accuracy by 5.59% compared with existing technologies, demonstrating high practical value. Yu et al. [5] studied the effects of aggregate texture, traffic volume, and rock characteristics on the decay of road surface friction coefficients, using various algorithms to build predictive models. The results indicate that traffic volume and rock type significantly affect friction decay, with the random forest algorithm demonstrating high predictive accuracy for large sample sizes. Jung et al. [6] investigated the effectiveness of an artificial neural network (ANN) classifier in estimating road surface friction coefficients based on field test data. By incorporating braking pressure and pressure gradient as inputs, they significantly improved estimation accuracy, particularly maintaining high accuracy under extreme road conditions. Tian et al. [7] proposed a fusion estimation method for tire–road peak friction coefficients (TRPAC) that accounts for model uncertainty. They designed an image fusion estimator based on virtual sensor theory and combined deep learning models with kinematic models for road condition classification. Experimental results show that this method offers significant advantages in estimation accuracy, convergence speed, and robustness compared with single-sensor estimation methods. Du et al. [8] proposed a computational model of a neural network simulating the structure and function of a biological neural network to estimate the road surface friction coefficient. Leng et al. [9] developed a fusion strategy combining dynamic and visual estimators to identify the road surface friction coefficient. These methods perform well in environments with adequate lighting and high visibility; however, their estimation accuracy significantly decreases when driving in nighttime conditions. To address the issue of inaccurate estimation during nighttime driving, some researchers have discovered that wheel deformation and vibrations are related to the road surface friction coefficient. Singh et al. [10] proposed a method for predicting the road surface friction coefficient using the frequency response of tire vibrations. Lampe et al. [11] used onboard sensors (such as a six-degrees-of-freedom inertial measurement unit, height-level sensors, and tie-rod force sensors) combined with the Unscented Kalman Filter algorithm to estimate peak friction coefficients. Experimental results indicated that model-based peak friction coefficient estimation using onboard sensors yielded good results. Schäfke et al. [12] proposed a method for estimating the maximum friction coefficient based on a transformer neural network (TNN) using onboard sensor data. Experimental results show that this method offers higher estimation accuracy and response speed, with fewer parameters and shorter training cycles. Yang et al. [13] proposed a strategy for improving the design of internal tire sensors to adapt to extreme conditions and enable continuous measurement of contact information. These methods require additional sensors to be installed on the vehicle, increasing costs, and the measurement accuracy is significantly affected by external interference, making it difficult to accurately measure the road surface friction coefficient.

Many scholars have conducted research on road surface friction coefficient estimation using vehicle dynamics principles and methods. Gauss et al. [14] first proposed a classical method for estimating the road surface friction coefficient using the longitudinal slip slope. This method requires fewer sensors and performs well, but issues with robustness and calibration have been identified. Therefore, the authors in [15,16] proposed nonlinear curve fitting techniques to address this problem. Yiğit [17] identified the road surface friction coefficient by examining the different slopes of the μ-s curve at small slip ratios. Kageyama et al. [18] proposed using the commonly applied magic tire formula in tire engineering to continuously measure the μ-s characteristics, enabling a more accurate measurement of the road surface friction coefficient. Lampe et al. [19] proposed a new method based on the Unscented Kalman Filter (UKF) to simultaneously estimate the maximum road friction coefficient, slope, and cross slope. By introducing a dynamic vehicle model and combining it with the UKF, they addressed the error issues associated with individual estimation, significantly improving estimation accuracy on roads with a slope or cross slope. Sun et al. [20] proposed an adaptive acceleration anti-skid control strategy based on tire slip ratio. Simulation results show that this method can rapidly and accurately estimate the friction coefficient on icy and snowy surfaces, effectively reducing shake and overshoot. Han et al. [21] proposed a method for estimating the tire–road peak friction coefficient (TRPFC), considering road roughness and texture. By optimizing the LuGre model to introduce the contact area ratio coefficient and combining the multi-point contact method and UKF, they achieved accurate TRPFC estimation. Both simulation and real-vehicle experiments validated the effectiveness of the algorithm. Xu et al. [22] proposed a method for estimating the three-dimensional road surface peak friction coefficient (TRPFC) based on a dimensionless data-driven tire model. They introduced a stress distribution and multi-point contact optimization model, combined with the UKF for real-time estimation, and fused longitudinal and lateral friction coefficients using fuzzy reasoning. Real-vehicle testing validated the method’s speed and accuracy.

Although the above methods can identify the road surface friction coefficient, onboard sensor-based methods require additional equipment on the vehicle, increasing costs. Furthermore, devices like cameras must operate under specific environmental conditions to effectively recognize the friction coefficient, limiting their practical application. Vehicle dynamics-based methods mostly do not account for vehicle nonlinearity and strong tire nonlinearity, making it difficult to accurately estimate the road surface friction coefficient at a slightly larger front wheel angle.

The Kalman filter algorithm can effectively achieve vehicle-related parameter identification. Vella et al. [23] used a linear Kalman filter algorithm to estimate the road profile and validated the algorithm through real vehicle experimental data. Galvagno et al. [24] achieved accurate measurement of the vehicle’s sideslip angle using only signals from onboard sensors through the Kalman filter algorithm. Reina et al. [25] utilized common onboard sensor signals and applied the Kalman filter algorithm to estimate the slip track. Considering that tires exhibit strong nonlinearity under a large front wheel angle, and that the Unscented Kalman Filter (UKF) is suitable for analyzing nonlinear problems, this paper, based on the practical needs of steer-by-wire systems, integrates the Dugoff tire model, AUKF, vehicle three-degrees-of-freedom dynamics model, and steer-by-wire actuator dynamics model to propose a piecewise estimation method for the road surface friction coefficient based on different front angle intervals within the large front wheel angle range. For a smaller front wheel angle (typically less than 3°), by real-time monitoring of the UKF divergence state through the covariance matching criterion, an adaptive weighting coefficient is designed to quickly correct the predicted covariance matrix. A noise estimator is constructed to dynamically update the system process noise parameters, and a forgetting factor is introduced to reduce the impact of outdated data on estimation accuracy. This optimization of the traditional Unscented Kalman Filter algorithm can effectively mitigate the impact of vehicle nonlinearity on the accuracy of road friction coefficient estimation. For a larger front wheel angle, considering the strong nonlinearity of the tires, the Adaptive Unscented Kalman Filter (AUKF) algorithm is not effective in estimating the road surface friction coefficient. Therefore, a rack force expansion state observer is used to estimate the rack force. Based on the principle that the rack force differs under different road surface friction coefficients at the same front wheel angle, the rack force is used to distinguish between different friction coefficients. Finally, the piecewise estimated road surface friction coefficient is compared with the corresponding friction coefficient in CarSim2019.1 to assess the accuracy of the proposed method.

## 2. Vehicle Dynamics Modeling

### 2.1. Tire Model

The Dugoff tire model [26] is selected for tire modeling due to its fewer required parameters and higher fitting accuracy. The longitudinal force Fx and lateral force Fy acting on each wheel can be expressed as

(1) $$Fx=\mu \cdot Fz\cdot Cx\cdot \frac{\lambda }{1-\lambda }\cdot f\left(L\right)$$

(2) $$Fy=\mu \cdot Fz\cdot Cy\cdot \frac{\tan(\alpha )}{1-\lambda }\cdot f\left(L\right)$$

(3) $$f\left(L\right)=\left\{\begin{array}{c} L(2-L),L<1\\ 1,L\geq 1 \end{array}\right.$$

(4) $$L=\frac{\left(1-\lambda \right)}{2\sqrt{C_{x}^{2}\cdot \lambda^{2}+C_{y}^{2}\cdot {\left(\tan\alpha \right)}^{2}}}$$

where μ is the road surface friction coefficient, Fz is the vertical force on the wheel, Cx is the longitudinal stiffness of the wheel, Cy is the lateral stiffness of the wheel, λ is the wheel slip ratio, and α is the wheel slip angle.

Simplifying the above equation,

(5) $$F_{x}^{0}=Fz\cdot Cx\cdot \frac{\lambda }{1-\lambda }\cdot f\left(L\right)$$

(6) $$F_{y}^{0}=Fz\cdot Cy\cdot \frac{\tan(\alpha )}{1-\lambda }\cdot f\left(L\right)$$

(7) $$Fx=\mu \cdot F_{x}^{0}$$

(8) $$Fy=\mu \cdot F_{y}^{0}$$

where $F_{x}^{0}$ and $F_{y}^{0}$ are the normalized expressions of the longitudinal and lateral forces of the tire, respectively. This form of expression facilitates the quick acquisition of the coefficient matrix when using Adaptive Unscented Kalman Filtering (AUKF) and improves the model’s accuracy in estimating the road surface friction coefficient.

Modeling the Dugoff tire model requires knowledge of the vertical force Fz on the wheel, the wheel slip angle α, and the wheel slip ratio λ. The specific calculation formulas can be referenced from relevant literature [27].

### 2.2. Vehicle Model

The relationship between the longitudinal and lateral forces of the four wheels and the road surface friction coefficient can be expressed as

(9) $$F_{xij}=\mu_{ij}\cdot F_{xij}^{0}\cdot \left(\lambda_{ij},\alpha_{ij},F_{zij}\right)$$

(10) $$F_{yij}=\mu_{ij}\cdot F_{yij}^{0}\cdot \left(\lambda_{ij},\alpha_{ij},F_{zij}\right)$$

where the subscripts *fr*, *fl*, *rr*, *rl* represent the right front wheel, left front wheel, right rear wheel, and left rear wheel, respectively.

**Figure 1:**
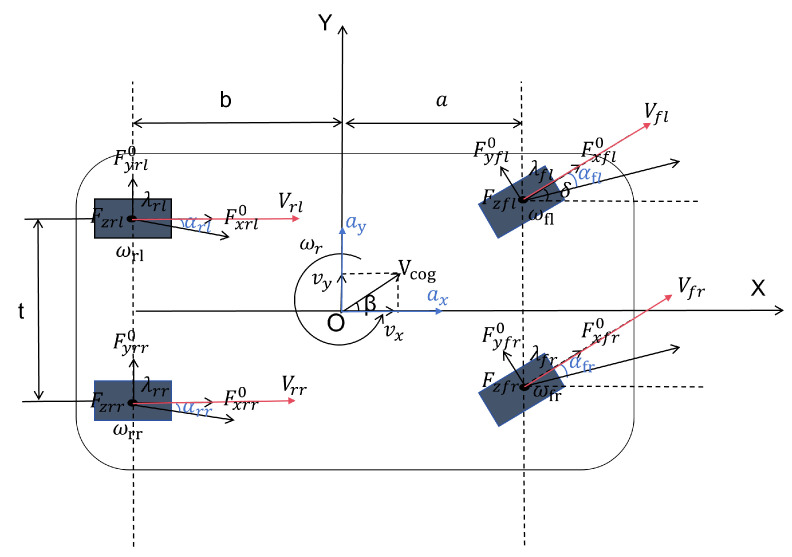
Three-degrees-of-freedom dynamics model of a four-wheel vehicle.

To simplify the analysis of vehicle motion, this paper constructs a four-wheel vehicle dynamics model, which focuses on studying the dynamic response of the vehicle in the three key directions: lateral, longitudinal, and yaw [28]. Figure 1 shows a schematic of the four-wheel vehicle dynamics model constructed in this paper, where *a* is the distance from the center of gravity to the front axle, *b* is the distance from the center of gravity to the rear axle, $a_{x}$ is the longitudinal acceleration, $a_{y}$ is the lateral acceleration, *h* is the center of gravity height, *L* is the distance from the front axle to the rear axle, t is the track width, $v_{x}$ is the longitudinal velocity, $v_{y}$ is the lateral velocity, δ is the front wheel angle, $w_{r}$ is the yaw rate, $w_{ij}$ represent the rotational speeds of the wheel, and $\alpha_{ij}$ represent the side slip angles of the wheel.

Based on the three-degrees-of-freedom dynamics model of the four-wheel vehicle, a force analysis is conducted for the vehicle in the lateral, longitudinal, and yaw directions, resulting in the following expressions:

(11) $$a_{x}=\frac{1}{m}\;\left(\mu_{fl}\cdot \left(F_{xfl}^{0}\cdot cos\delta -F_{yfl}^{0}\cdot sin\delta \right)+\mu_{fr}\cdot \left(F_{xfr}^{0}\cdot cos\delta -F_{yfr}^{0}\cdot sin\delta \right)+\mu_{rl}\cdot F_{xrl}^{0}+\mu_{rr}\cdot F_{xrr}^{0}\right)$$

(12) $$a_{y}=\frac{1}{m}\left(\mu_{fl}\cdot \left(F_{xfl}^{0}\cdot sin\delta +F_{yfl}^{0}\cdot cos\delta \right)+\mu_{fr}\cdot \left(F_{xfr}^{0}\cdots in\delta +F_{yfr}^{0}\cdot sin\delta \right)+\mu_{rl}\cdot F_{yrl}^{0}+\mu_{rr}\cdot F_{yrr}^{0}\right)$$

(13) $$\begin{array}{cc} \; & {\dot{\omega }}_{r}=\frac{1}{I_{z}}\cdot (a\cdot \left(F_{xfl}^{0}\cdot sin\delta +F_{yfl}^{0}\cdot cos\delta \right)\cdot \mu_{fl}+a\cdot \left(F_{xfr}^{0}\cdot sin\delta +F_{yfr}^{0}\cdot cos\delta \right)\cdot \mu_{fr}+\\ \; & \frac{t}{2}\cdot \left(-F_{xfl}^{0}\cdot cos\delta +F_{yfl}^{0}\cdot cos\delta \right)\cdot \mu_{fl}+\frac{t}{2}\cdot \left(F_{xfr}^{0}\cdot cos\delta -F_{yfr}^{0}\cdot sin\delta \right)\cdot \mu_{fr}-b\cdot \mu_{rl}\cdot F_{yrl}^{0}\\ \; & -\frac{t}{2}\cdot \mu_{rl}\cdot F_{xrl}^{0}-b\cdot \mu_{rr}\cdot F_{yrr}^{0}+\frac{t}{2}\cdot \mu_{rr}\cdot F_{xrr}^{0}) \end{array}$$

where Iz is the moment of inertia, $F_{yij}^{0}$ is the lateral normalized tire force, and $F_{xij}^{0}$ is the longitudinal normalized tire force.

## 3. Piecewise Combination Estimation of Road Surface Friction Coefficient Based on Dynamics Model Fusion

### 3.1. Principle of Adaptive Unscented Kalman Filtering

The design of UKF depends on an accurate mathematical model. If there are significant deviations in the dynamic model or mismatches in noise characteristics, the estimation accuracy may degrade significantly, leading to divergence in the observed results. To address the divergence issue of UKF under noise influence, the covariance matching criterion is employed to monitor the divergence state of the Unscented Kalman Filter (UKF) in real time [29]. Additionally, a noise estimator with a forgetting factor is introduced to dynamically update the process noise parameters, and an adaptive weighting coefficient is applied to correct the predicted covariance matrix [30]. This leads to the development of the Adaptive Unscented Kalman Filter (AUKF) algorithm. With its precise approximation of nonlinear systems and efficient computational performance, the Adaptive Unscented Kalman Filter provides a reliable state estimation method for automotive system dynamics analysis.

#### Basic Process of AUKF (Adaptive Unscented Kalman Filter)

Due to the possibility of the filter diverging as a result of inaccurate data updates from the observer, this paper introduces an innovation covariance matching principle to determine whether the filtering process is diverging. The principle is as follows:

(14) $${\tilde{y}}_{k+1}^{T}{\tilde{y}}_{k+1}>\zeta tr\left[E\left({\tilde{y}}_{k+1}{\tilde{y}}_{k+1}^{T}\right)\right]$$

(15) $${\tilde{y}}_{k+1}=y_{k+1}-{\hat{y}}_{k+1|k}$$

where ${\tilde{y}}_{k+1}^{T}$ is the innovation covariance at time *k* + 1, tr(·) denotes the trace of a matrix, and ζ>1 is the adjustable coefficient.

If Equation (23) holds, it indicates that the actual error has exceeded ζ times the threshold. Therefore, this paper sets the adaptive weighting coefficient $\rho_{k}$ to correct the predicted variance, as shown below:

(16) $$P\left(k|k-1\right)=\rho_{k}\sum_{i=0}^{2N}W_{i}^{\left(c\right)}\left(\chi_{i}\left(k|k-1\right)-\hat{x}\left(k|-1\right)\right){\left(\chi_{i}\left(k|k-1\right)-\hat{x}\left(k|k-1\right)\right)}^{T}+Q_{k}$$

where

(17) $$\rho_{k}=\left\{\begin{array}{cc} \rho_{0} & \rho_{0}\geq 1\\ 1 & \rho_{0}<1 \end{array}\right.$$

(18) $$\rho_{0}=\frac{tr{\left(N_{0,k+1}-R_{k}\right)}^{T}}{tr{\left(P_{X_{k}Z_{k}}\right)}^{T}}$$

(19) $$N_{0,k+1}=\left\{\begin{array}{c} {\tilde{y}}_{k+1}{\tilde{y}}_{k+1}^{T}\\ \frac{\partial N_{0,k}+{\tilde{y}}_{k+1}{\tilde{y}}_{k+1}^{T}}{1+\partial } \end{array}\right.$$

where *∂* is the decay coefficient, where 0<∂<1, typically taken as 0.95 to enhance the filter’s convergence ability.

Perform measurement update, where the observation values are multiplied by the weights and summed to obtain the predicted mean and covariance of the system. Calculate the updated state covariance.

(20) $$P_{yy}=\sum_{i=0}^{2N}W_{i}^{\left(c\right)}\left(y_{k+1|k}^{i}-{\hat{y}}_{k+1|k}\right){\left(y_{k+1|k}^{i}-{\hat{y}}_{k+1|k}\right)}^{T}+R_{k}$$

Calculate the cross-covariance matrix.

(21) $$P_{xy}\left(k|k-1\right)=\sum_{i=0}^{2N}W_{i}^{\left(c\right)}\left(y_{k+1|k}^{i}-{\hat{y}}_{k+1|k}\right){\left(y_{k+1|k}^{i}-{\hat{y}}_{k+1|k}\right)}^{T}$$

Calculate the updated filter feedback gain.

(22) $$K\left(k\right)=P_{xy}\left(k|k-1\right)P_{yy}^{-1}$$

Calculate the filtered value after state update.

(23) $$\hat{x}\left(k|k\right)=\hat{x}\left(k|k-1\right)+K\left(k\right)\left(y_{k+1|k}-{\hat{y}}_{k+1|k}\right)$$

Calculate the posterior state covariance matrix.

(24) $$P\left(k|k\right)=P\left(k|k-1\right)-K\left(k\right)P_{yy}K{\left(k\right)}^{T}$$

To address the unknown nature of system time-varying noise, this paper uses the Sage–Husa estimator [31] to estimate the mean ${\hat{q}}_{k}$ and covariance ${\hat{Q}}_{k}$ of the system process noise in real time, with the following expression:

(25) $${\hat{q}}_{k}=k^{-1}\sum_{j=0}^{k}\left[{\hat{X}}_{j+1|j+1}-\sum_{i=0}^{2n}\omega_{i}^{\left(c\right)}\chi_{j|j}^{i}\right]$$

(26) $${\hat{Q}}_{k}=k^{-1}\sum_{j=0}^{k}\left[\begin{array}{c} \left({\hat{X}}_{j+1|j+1}-{\hat{X}}_{j+1|j}\right){\left({\hat{X}}_{j+1|j+1}-{\hat{X}}_{j+1|j}\right)}^{T}+P_{j+1|j+1}-{\hat{P}}_{j+1|j} \end{array}\right]$$

This paper introduces a forgetting factor to emphasize the role of recent data, so the new expression is

(27) $${\hat{q}}_{k}=k^{-1}\sum_{j=0}^{k}g_{j}\left[{\hat{X}}_{j+1|j+1}-\sum_{i=0}^{2n}\omega_{i}^{\left(c\right)}\chi_{j|j}^{i}\right]$$

(28) $${\hat{Q}}_{k}=k^{-1}\sum_{j=0}^{k}g_{j}\left[\begin{array}{c} \left({\hat{X}}_{j+1|j+1}-{\hat{X}}_{j+1|j}\right){\left({\hat{X}}_{j+1|j+1}-{\hat{X}}_{j+1|j}\right)}^{T}+P_{j+1|j+1}-{\hat{P}}_{j+1|j} \end{array}\right]$$

where $g_{j}$ satisfies

(29) $$\left\{\begin{array}{c} g_{j}=d_{k}c^{k-j}\\ \sum_{j=1}^{k}g_{j}=1 \end{array}\right.$$

where *g* is the forgetting factor, and $d_{k}=\left(1-c\right)/\left(1-c^{k}\right)$.

By substituting Equation (29) into Equations (27) and (28), the expressions for the mean ${\hat{q}}_{k}$ and covariance ${\hat{Q}}_{k}$ are derived as

(30) $$\begin{array}{c} {\hat{q}}_{k}=\left(1-\frac{1-c}{1-c^{k}}\right){\hat{q}}_{k-1}+\frac{1-c}{1-c^{k}}\left[{\hat{X}}_{k|k}-\sum_{i=0}^{2n}\omega_{i}^{\left(c\right)}\chi_{k-1|k-1}^{i}\right] \end{array}$$

(31) $${\hat{Q}}_{k}=\left(1-\frac{1-c}{1-c^{k}}\right){\hat{Q}}_{k-1}+\frac{1-c}{1-c^{k}}\left\{\begin{array}{c} \left[{\hat{X}}_{k|k}-{\hat{X}}_{k|k-1}\right]{\left[{\hat{X}}_{k|k}-{\hat{X}}_{k|k-1}\right]}^{T}+P_{k|k}-{\hat{P}}_{k|k-1} \end{array}\right\}$$

**Figure 2:**
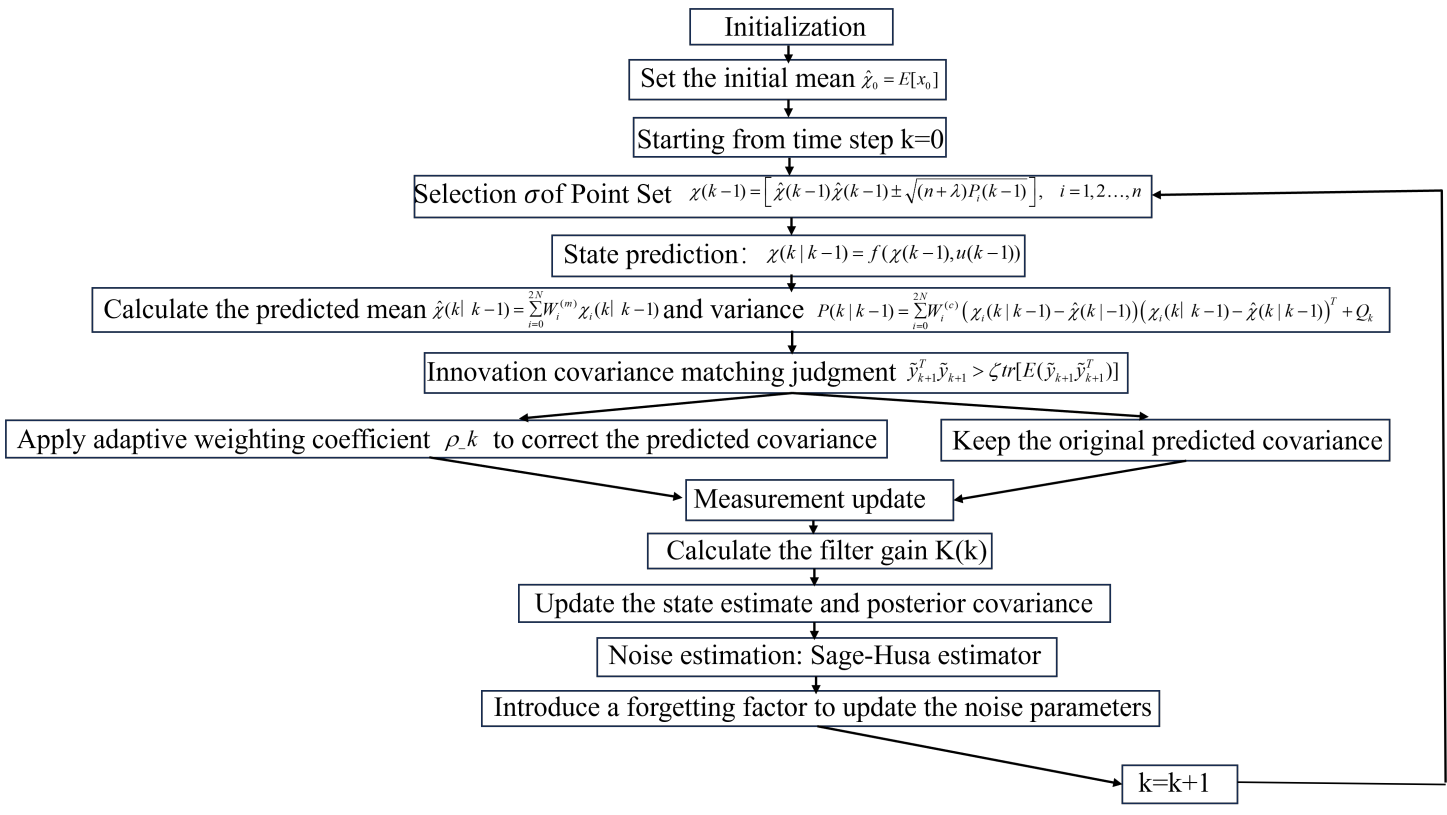
Steps of the Adaptive Unscented Kalman Filter (AUKF) algorithm.

The process of the adaptive unscented Kalman filter algorithm is shown in Figure 2:

### 3.2. Road Surface Friction Coefficient Estimation Based on Adaptive Unscented Kalman Filtering

Using the dynamic equations established by the above three-degrees-of-freedom model, as shown in Equations (24)–(26), combined with the data measured by sensors, the system measurement equation is set as y(t), which includes longitudinal acceleration, lateral acceleration, and yaw acceleration, while the system state equation is x(t), representing the road surface friction coefficient for the four wheels, and the control variable is the front wheel angle $\left[\delta \right]$.

By linking the above vehicle dynamics equations, the filter’s state equation can be written in the following form:

(32) $$\dot{x}\left(t\right)=\left[\begin{array}{cccc} 1 & 0 & 0 & 0\\ 0 & 1 & 0 & 0\\ 0 & 0 & 1 & 0\\ 0 & 0 & 0 & 1 \end{array}\right]\left[\begin{array}{c} \mu_{fl}\\ \mu_{fr}\\ \mu_{rl}\\ \mu_{rr} \end{array}\right]+w\left(t\right)$$

The filter’s measurement equation can also be written in the following form:

(33) $$y\left(t\right)=\left[\begin{array}{cccc} \frac{F_{xfl}^{0}\cdot cos\delta -F_{yfl}^{0}\cdot sin\delta }{m} & \frac{F_{xfr}^{0}\cdot cos\delta -F_{yfr}^{0}\cdot sin\delta }{m} & \frac{F^{0}xrl}{m} & \frac{F^{0}xrr}{m}\\ \frac{F_{xfl}^{0}\cdot sin\delta +F_{yfl}^{0}\cdot cos\delta }{m} & \frac{F_{xfr}^{0}\cdot sin\delta +F_{yfr}^{0}\cdot cos\delta }{m} & \frac{F^{0}yrl}{m} & \frac{F^{0}yrr}{m}\\ h(3,1) & h(3,2) & h(3,3) & h(3,4) \end{array}\right]\left[\begin{array}{c} \mu_{fl}\\ \mu_{fr}\\ \mu_{rl}\\ \mu_{rr} \end{array}\right]+\nu \left(t\right)$$

where:

(34) $$h\left(3,1\right)=\frac{a\left(F_{xfl}^{0}\cdot sin\delta +F_{yfl}^{0}\cdot cos\delta \right)-\frac{t}{2}\left(F_{xfl}^{0}\cdot cos\delta -F_{yfl}^{0}\cdot sin\delta \right)}{I_{z}}$$

(35) $$h\left(3,2\right)=\frac{a\left(F_{xfr}^{0}\cdot sin\delta +F_{yfr}^{0}\cdot cos\delta \right)+\frac{t}{2}\left(F_{xfl}^{0}\cdot cos\delta -F_{yfr}^{0}\cdot sin\delta \right)}{I_{z}}$$

(36) $$h\left(3,3\right)=\frac{-\frac{t}{2}\cdot F^{0}xrl-b\cdot F^{0}yfl}{I_{z}}$$

(37) $$h\left(3,4\right)=\frac{\frac{t}{2}\cdot F^{0}xrr-b\cdot F^{0}yrr}{I_{z}}$$

After multiple adjustments, the noise in the state estimation process w(t) and the observation noise v(t) are set, and it follows that

(38) $$\left\{\begin{array}{c} E\left[W_{k}\right]=q_{k},cov\left[w_{k},w_{j}^{T}\right]=Q_{k}\\ E\left[V_{k}\right]=r_{k},cov\left[v_{k},v_{j}^{T}\right]=R_{k} \end{array}\right.$$

In the formula, $q_{k}$ and $r_{k}$ are the non-zero means of $W_{k}$ and $V_{k}$ at time *k*, and $Q_{k}$ and $R_{k}$ are the covariances of $W_{k}$ and $V_{k}$ at time *k*. The error covariance matrix $P\left(t0\right)=I_{4*4}$. The initial value of $X_{o}$ is set to $\left[\begin{array}{c} 0,0,0,0 \end{array}\right]$.

### 3.3. Road Surface Friction Coefficient Estimation Based on Steering Rack Force

#### 3.3.1. Steering Rack Force Observer Design

When the vehicle’s front wheel angle is large (typically greater than 3°), the tires enter the nonlinear region, causing the tire force estimated by the Dugoff tire model to have larger errors, which leads to the failure of the Adaptive Unscented Kalman Filtering algorithm for road surface friction coefficient estimation. Therefore, this section uses the extended observer method to estimate the steering rack force. Based on the principle that the steering rack force is different for different road surface friction coefficients at the same front wheel angle, the road surface friction coefficient can be distinguished through the steering rack force.

**Figure 3:**
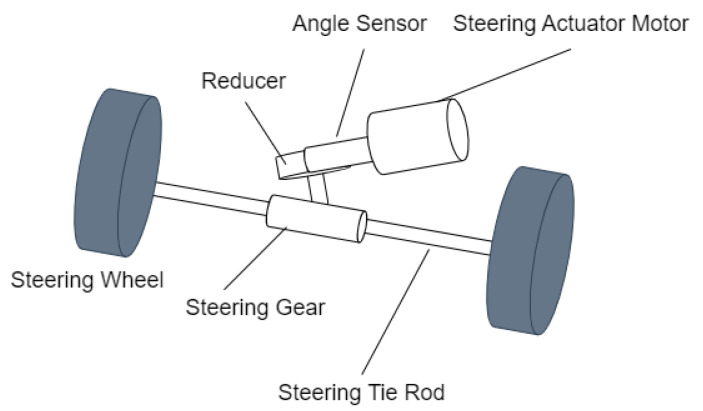
Steer-by-wire actuator module.

To observe the steering rack force, it is first necessary to establish the dynamic model of the steer-by-wire actuator, as shown in Figure 3. Given that the motor shaft rotation friction is very small, the modeling process neglects the effect of motor shaft rotational friction. The dynamic equation is as follows:

(39) $$\left\{\begin{array}{c} J_{m}{\ddot{\theta }}_{m}+B_{m}{\dot{\theta }}_{m}+K_{m}\left(\frac{\theta_{m}}{G_{m}}-\frac{X_{r}}{r_{p}}\right)=T_{m}\\ m_{r}{\ddot{X}}_{r}+B_{r}{\dot{X}}_{r}+F_{e}=K_{m}\left(\frac{\theta_{m}}{G_{m}}-\frac{X_{r}}{r_{p}}\right)\cdot \frac{G_{m}}{r_{p}} \end{array}\right.$$

where $J_{m}$ is the motor moment of inertia, $B_{m}$ is the motor viscous damping coefficient, $K_{m}$ is the drive shaft torsional stiffness, $G_{m}$ is the gearbox reduction ratio, $r_{p}$ is the pitch circle radius, and $\theta_{m}$ is the motor rotor angle. $m_{r}$ is the rack mass, $B_{r}$ is the rack damping, $F_{e}$ is the steering rack force, and $X_{r}$ is the rack displacement.

The authors in [32] discuss that the extended observer, compared with linear or nonlinear disturbance observers, provides better performance in estimating the steering rack force when the steering system is under extreme operating conditions. Therefore, this paper uses an extended observer to estimate the steering rack force.

First, define $x_{1}=\theta_{m},x_{2}=\dot{\theta_{m}},x_{3}=X_{r},x_{4}=\dot{X_{r}}$ as the system state variables, and define the extended state $x_{5}$ as the steering rack force $F_{e}$. In addition, the signals that need to be measured are the motor angle and the steering rack displacement, both of which can be measured using sensors, and are defined as $y_{1}$ and $y_{2}$, respectively.

Based on Equation (39), the extended state observer is designed as

(40) $$\begin{array}{cc} \; & {\dot{\hat{x}}}_{1}={\hat{x}}_{2}+Z_{1}\left(y_{1}-{\hat{x}}_{1}\right)\\ \; & {\dot{\hat{x}}}_{2}=-\frac{K_{m}}{G_{m}J_{m}}{\hat{x}}_{1}-\frac{B_{m}}{J_{m}}{\hat{x}}_{2}+\frac{K_{m}}{J_{m}r_{p}}{\hat{x}}_{3}+Z_{2}\left(y_{1}-{\hat{x}}_{1}\right)+\frac{1}{J_{m}}T_{m}\\ \; & {\dot{\hat{x}}}_{3}={\hat{x}}_{4}+Z_{3}\left(y_{2}-{\hat{x}}_{3}\right)\\ \; & {\dot{\hat{x}}}_{4}=\frac{K_{m}}{r_{p}m_{r}}{\hat{x}}_{1}-\frac{K_{m}G_{m}}{r_{p}^{2}m_{r}}{\hat{x}}_{3}-\frac{1}{m_{r}}{\hat{x}}_{5}+Z_{4}\left(y_{2}-{\hat{x}}_{3}\right)\\ \; & {\dot{\hat{x}}}_{5}=-Z_{5}\left(y_{1}-{\hat{x}}_{1}\right)-Z_{6}\left(y_{2}-{\hat{x}}_{3}\right) \end{array}$$

where ${\dot{\hat{x}}}_{1},{\dot{\hat{x}}}_{2},{\dot{\hat{x}}}_{3},{\dot{\hat{x}}}_{4},{\dot{\hat{x}}}_{5}$ are the estimated values of the state variables $x_{1},x_{2},x_{3},x_{4},x_{5}$, where $Z_{1},Z_{2},Z_{3},Z_{4},Z_{5}$ are the feedback gains of the observer, which need to be designed [33].

(41) $$Z_{1}=\frac{\alpha_{1}}{\varepsilon },Z_{2}=\frac{\alpha_{2}}{\varepsilon^{2}},Z_{3}=\frac{\beta_{1}}{\varepsilon },Z_{4}=\frac{\beta_{2}}{\varepsilon^{2}},Z_{5}=\frac{\alpha_{3}}{\varepsilon^{3}},Z_{6}=\frac{\beta_{3}}{\varepsilon^{3}}$$

Equation (41) serves for parameter tuning of the rack force expansion observer in Equation (40). The parameters $\alpha_{1},\alpha_{2},\alpha_{3},\beta_{1},\beta_{2},\beta_{3},\varepsilon$ need to be designed.The control input of the observer is the motor torque $T_{m}$, and the steering rack force is observed as the system disturbance.

The main parameters of the steering-by-wire system actuator structure are shown in Table 1.

#### 3.3.2. Road Surface Friction Coefficient Estimation Based on Steering Rack Force

**Figure 4:**
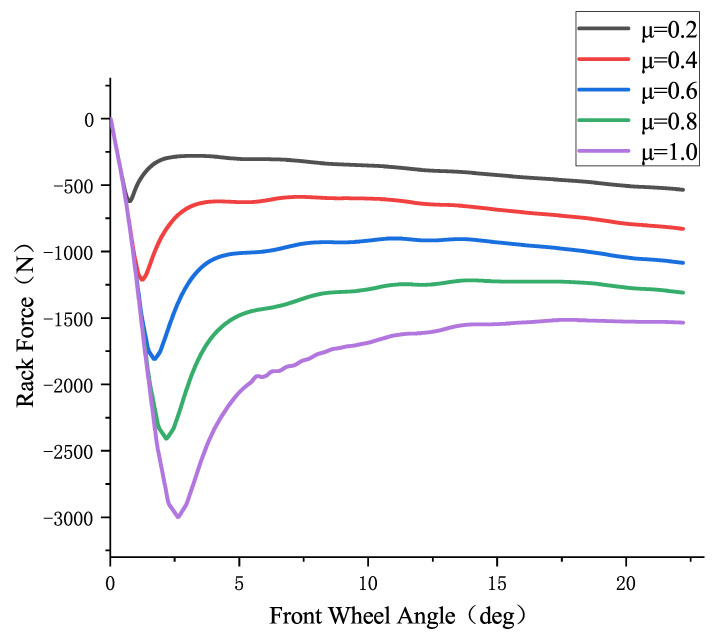
Relationship between front wheel angle and steering rack force at a vehicle speed of 60 km/h.

From the steering rack force observation model in the previous section, the relationship between the front wheel angle and the steering rack force is shown in Figure 4 as follows:

Figure 4 shows the relationship curves between steering rack force and front wheel angle at different road surface friction coefficients (0.2, 0.4, 0.6, 0.8, 1.0). These curves are estimated using the steering rack force observer described in the previous section. From Figure 4, it can be observed that when the front wheel angle is less than 2.2°, the steering rack force is almost identical for different friction coefficients at the same front wheel angle. Therefore, it is not possible to distinguish different road surface friction coefficients within this range using the steering rack force. However, when the front wheel angle is greater than 2.2°, the steering rack force shows a significant difference for different road surface friction coefficients at the same front wheel angle, allowing for effective differentiation between various friction coefficients.

In practical testing, the front wheel angle δ can be obtained using a steering angle sensor, and the steering rack force F can be estimated in real time using the steering rack force extended state observer. Then, by using the front wheel angle δ as input, a lookup table module can be used to obtain the steering rack force values for different road surface friction coefficients: the rack force F1 for a friction coefficient of 0.2, F2 for 0.4, F3 for 0.6, F4 for 0.8, and F5 for 1.0. By checking where the real-time steering rack force F lies within the range between F1 and F5, the current road surface friction coefficient range can be inferred. For example, if F is between F2 and F3, it indicates that the road surface friction coefficient is between 0.4 and 0.6. Based on the judgment logic designed in this paper, the road surface friction coefficient can further be determined to be 0.5.

Figure 4 also shows the relationship curve between steering rack force and front wheel angle at a vehicle speed of 60 km/h. Since, in real driving conditions, vehicle speed is not constant at a specific value, this paper divides the speed range from 0 to 120 km/h into intervals of 5 km/h, and estimates the steering rack force for each speed range using the steering rack force observer designed in Section 3.3.1. A lookup table module is then constructed with the front wheel angle as the horizontal axis and the steering rack force as the vertical axis. By selecting the lookup table corresponding to the speed closest to the real vehicle speed, the steering rack force can be estimated across the entire vehicle speed range. For example, when the actual vehicle speed is 58 km/h, it is closest to 60 km/h, so the lookup table based on 60 km/h is used for the estimation.

## 4. Simulation Experiment and Results Analysis

### 4.1. Estimation of Road Surface Friction Coefficient Based on the Combination of AUKF Algorithm and Rack Force Method

To verify the accuracy of the road surface friction coefficient estimation using the Adaptive Unscented Kalman Filter algorithm and rack force method, simulation experiments were conducted with high and low road surface friction coefficients set in the CarSim software. The estimated results from the algorithm were compared with the preset friction coefficients in CarSim to evaluate the effectiveness of the algorithm.

#### 4.1.1. Joint Simulation of CarSim and Matlab/Simulink

To verify the accuracy of the proposed algorithm for road surface friction coefficient estimation, this paper uses CarSim software to receive front wheel angle and vehicle speed signals, and outputs the wheel speed signals required by the Adaptive Unscented Kalman Filtering algorithm, including yaw rate, longitudinal acceleration, lateral acceleration, center of gravity slip angle, and longitudinal vehicle speed. Then, the vehicle’s three-degrees-of-freedom model and Dugoff normalized tire model are built using Matlab/Simulink. At the start of the simulation, initial conditions are set, and various typical road surfaces are selected for testing. Finally, the road surface friction coefficients estimated by the Adaptive Unscented Kalman Filtering algorithm and rack force are compared with the corresponding road surface friction coefficients in CarSim software to assess the accuracy of the proposed algorithm. The vehicle parameters are shown in Table 2.

#### 4.1.2. Results Analysis

**Figure 5:**
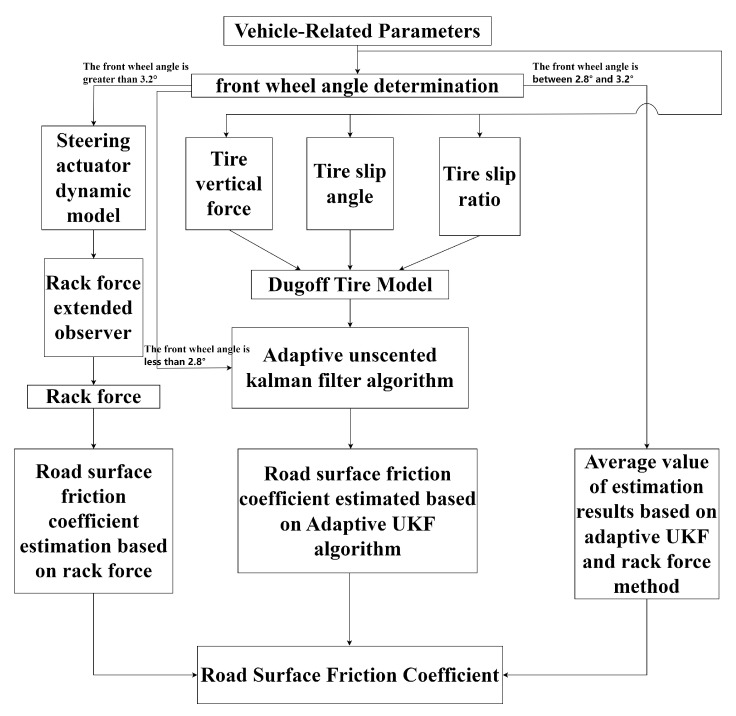
Segmented estimation scheme for road surface friction coefficient.

The road surface friction coefficient can be accurately estimated using the AUKF algorithm when the front wheel angle is small. However, when the front wheel angle exceeds 3.89°, the estimation results no longer fluctuate within a small range around the set road surface friction coefficient, leading to the failure of the method. In contrast, the rack force method estimates the road surface friction coefficient well when the front wheel angle is large. When the front wheel angle is small, the estimated rack forces for different road surface friction coefficients are equal, making it impossible to estimate the friction coefficient accurately. However, when the front wheel angle exceeds 2.78°, the method can estimate the road surface friction coefficient with reasonable accuracy.It is evident that the two estimation methods complement each other. By combining both methods, a high-precision estimation of the road surface friction coefficient across the entire range of front wheel angles can be achieved. When the front wheel angle is less than 2.78°, the road surface friction coefficient is estimated using the AUKF algorithm. When the front wheel angle exceeds 3.2°, the friction coefficient is estimated based on the rack force method. For front angles between 2.78° and 3.2°, both the AUKF algorithm and the rack force method can estimate the road surface friction coefficient, but it may be difficult to distinguish between their respective accuracies. Considering the feasibility for practical applications, this paper proposes using the average of the estimation results from both methods as the estimated road surface friction coefficient in this range of front wheel angles. Therefore, a segmented estimation scheme for the road surface friction coefficient, as shown in Figure 5, is proposed.

**Figure 6:**
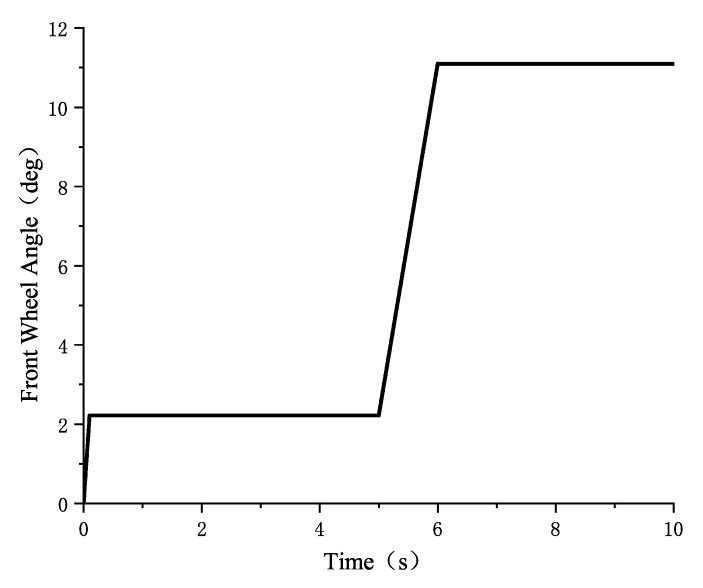
Front wheel angle input.

**Figure 7:**
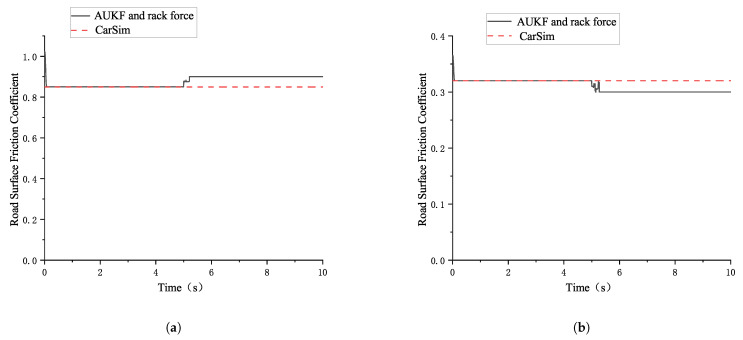
The simulation results for the road surface friction coefficient estimation based on the combination of AUKF and rack force. (**a**) Segmented road surface friction coefficient estimation simulation result (large front wheel angle high-friction coefficient). (**b**) Segmented road surface friction coefficient estimation simulation result (large front wheel angle low-friction coefficient).

In the CarSim simulation, the vehicle driving condition parameters are set as follows: the road surface friction coefficients are set to 0.85 and 0.32, respectively, and the vehicle speed is 50 km/h, with the front wheel angle input set to a step input from 0° to 2.22°, followed by a step input from 2.22° to 11.11°. The front wheel angle input is shown in Figure 6:

The simulation results for the road surface friction coefficient estimation based on the combination of AUKF and rack force are shown in the following Figure 7:

As seen in Table 3, whether for high- or for low-friction coefficient road surfaces, the estimated road surface friction coefficient based on the combination of the AUKF algorithm and the rack force method shows small absolute and relative errors compared with the road surface friction coefficient set in CarSim. This method effectively estimates the road surface friction coefficient with good results.

### 4.2. Comparison with the Unscented Kalman Filter Method Results

To validate the effectiveness of the proposed segmented estimation method based on multimodal vehicle dynamics fusion, compared with the traditional Unscented Kalman Filter (UKF) method for road surface friction coefficient estimation, simulation experiments were conducted in the CarSim software. The high and low road surface friction coefficients were set for the road conditions. Both the segmented estimation method and the UKF algorithm were used to estimate the road surface friction coefficient, and the results were compared with the road surface friction coefficients set in CarSim. This comparison was performed to analyze the estimation accuracy of the two methods.

**Figure 8:**
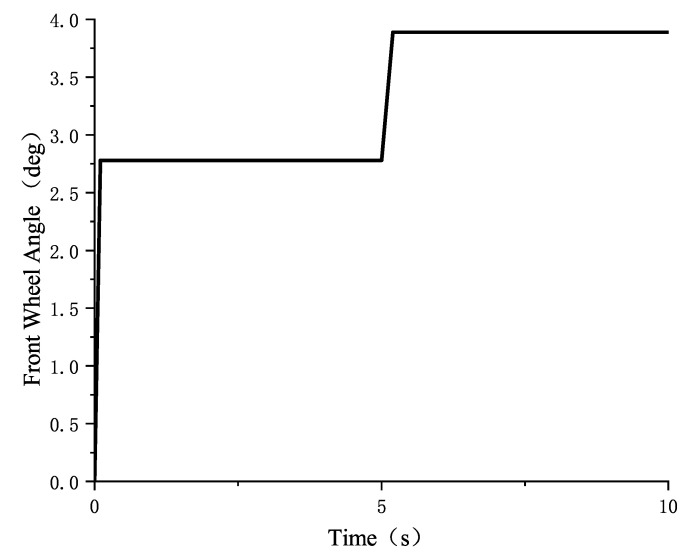
Front wheel angle input from small to large.

**Figure 9:**
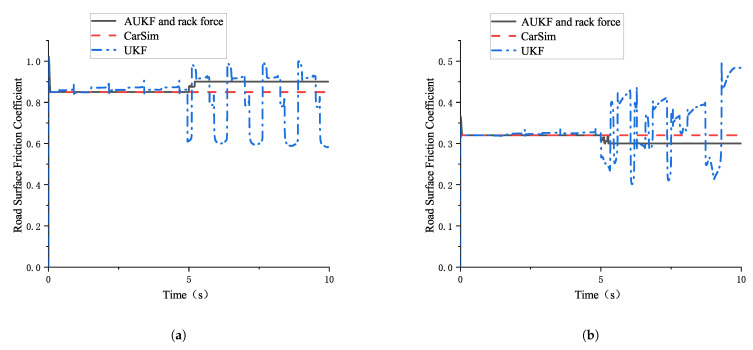
Estimation results for road surface friction coefficient based on the combination of AUKF and rack force method and UKF algorithm. (**a**) Comparison of estimation result based on the AUKF and rack force method and the UKF algorithm (high-friction coefficient). (**b**) Comparison of estimation result based on the AUKF and rack force combined method and the UKF algorithm (low-friction coefficient).

In the CarSim simulation, the vehicle operating parameters are set as follows: the road surface friction coefficients are set to 0.85 and 0.32, respectively; the vehicle speed is 50 km/h; and the front wheel angle is set to a step input from 0° to 2.78°, followed by a step input from 2.78° to 3.89°. The front wheel angle input is shown in the following Figure 8:

The estimation results of the road surface friction coefficient based on the AUKF and rack force method proposed in this paper, compared with the results based on the UKF algorithm, are shown in Figure 9:

The simulation results show that, across the entire steering angle range, whether under high- or low-friction conditions, the absolute and relative errors between the road surface friction coefficient estimated using the AUKF algorithm and rack force method and the road surface friction coefficient set in CarSim are smaller than those of the UKF algorithm. Clearly, the accuracy of estimating high-friction coefficients using the AUKF algorithm and rack force method is significantly higher than that of the UKF algorithm.

## 5. HIL Testing of the Steer-by-Wire System

In the previous chapter, the Adaptive Unscented Kalman Filter (AUKF) algorithm designed in this paper, combined with a rack force-based method for road surface friction coefficient estimation, was validated through joint simulations using Matlab/Simulink and CarSim. However, the simulation was conducted in a relatively ideal environment, neglecting the effects of signal transmission delays and noise between components and simplifying the dynamic model of the steer-by-wire actuator in CarSim. To further validate the feasibility of the proposed algorithm, this chapter conducts tests using a hardware-in-the-loop (HIL) test rig for the steer-by-wire system.

### 5.1. Composition and Functions of the Steer-by-Wire System HIL Test Rig

**Figure 10:**
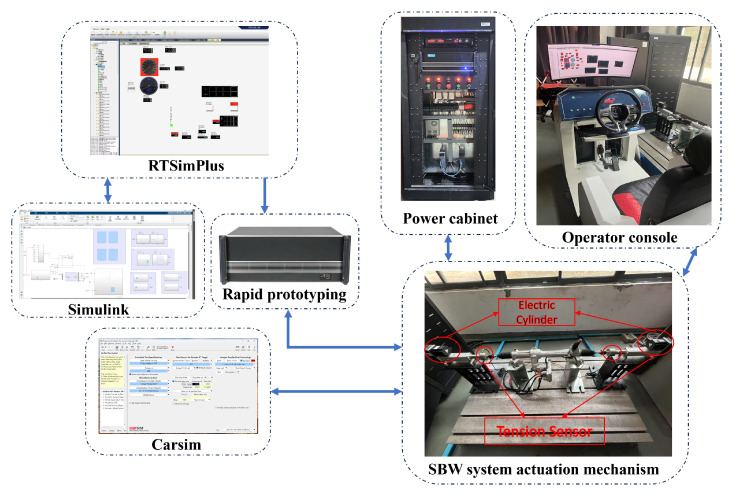
Steer-by-wire hardware-in-the-loop test rig.

The steer-by-wire system HIL test rig adopts a modular design. The hardware consists of modules such as the steer-by-wire test bench, road condition simulation load system, and power distribution cabinet. The software includes the vehicle dynamics software CarSim 2021.0, the mathematical computation software Matlab 2022a, and the real-time simulation testing software RTSimPlus 2019, among others. The modules can be used independently or in combination. The various sensors in the test rig can collect relevant parameters in real time and enable communication between components via CAN bus cards. It supports functions and performance tests such as road surface friction coefficient estimation, rack force observation, human–machine co-driving, and road feel simulation. The steer-by-wire system HIL test rig is shown in the following Figure 10:

### 5.2. HIL Test of Road Surface Friction Coefficient Estimation Based on AUKF Algorithm and Rack Force Method

**Figure 11:**
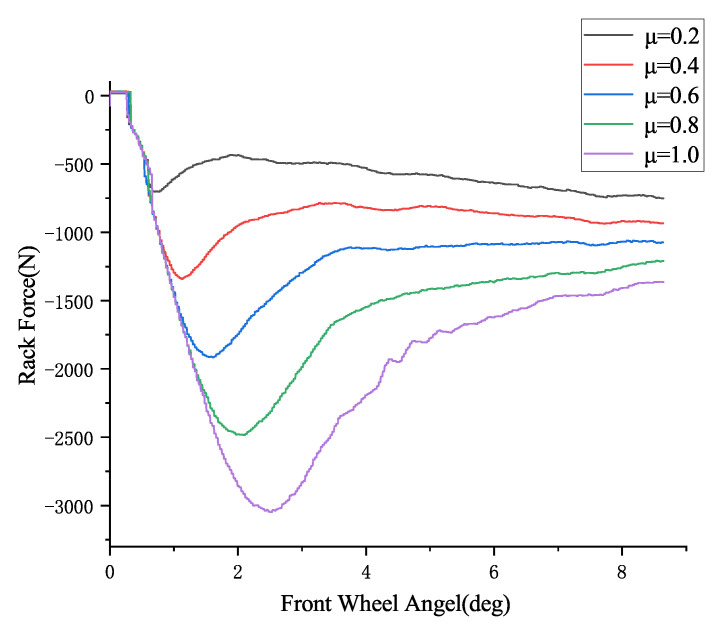
Relationship between front wheel angle and rack force at a vehicle speed of 50 km/h.

During the hardware-in-the-loop (HIL) test, the rack force under different road surface friction coefficients is first measured using the force sensor on the steer-by-wire system actuator. Then, the relationship between front wheel angle and rack force at different vehicle speeds is used to create a corresponding lookup table module. Figure 11 shows the relationship between front wheel angle and the measured rack force at a vehicle speed of 50 km/h. The rack force measured by the force sensor also shows that, under conditions where the front wheel angle is less than 2.4°, the rack forces for different friction coefficients are almost identical at the same front wheel angle. Therefore, within this range, it is not possible to distinguish between different road surface friction coefficients based on the rack force. When the front wheel angle exceeds 2.4°, under the same front wheel angle condition, there is a significant difference in rack force for different road surface friction coefficients, enabling effective differentiation of the road surface friction coefficients.

**Figure 12:**
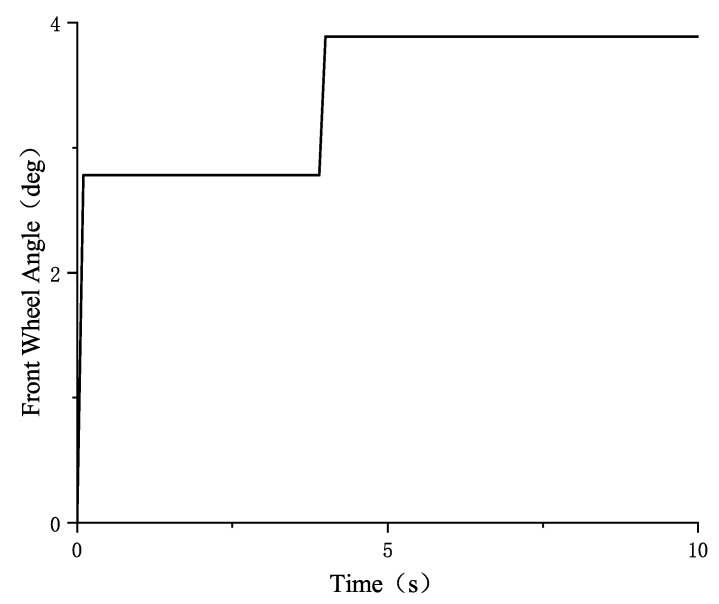
Front wheel angle input from small to large.

In CarSim, the vehicle operating conditions are set as follows: the road surface friction coefficient is set to 0.85 and 0.32, the vehicle speed is 50 km/h, and the front wheel angle is set as a step input from 0° to 2.78°, followed by another step input from 2.78° to 3.89°. The front wheel angle input is shown in Figure 12.

**Figure 13:**
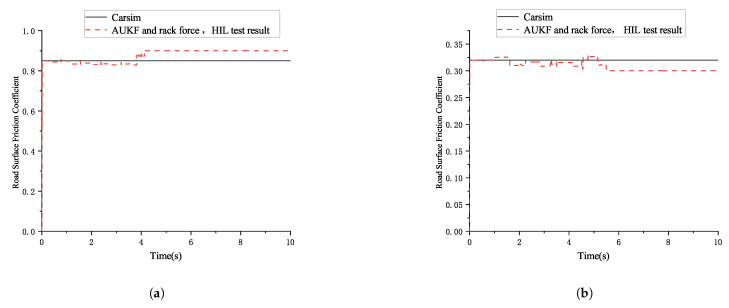
The HIL test results for the road surface friction coefficient estimation based on the combination of AUKF and rack force. (**a**) HIL test result for road surface friction coefficient estimation (large front wheel angle high-friction coefficient). (**b**) HIL test result for road surface friction coefficient estimation (large front wheel angle low-friction coefficient).

The HIL test results for the road surface friction coefficient estimation based on the combination of AUKF and rack force are shown in the following Figure 13:

As shown in Table 4, under the high-friction and small-steering-angle condition, the absolute error between the semi-physical test results of road friction coefficient estimation, based on the combination of the AUKF algorithm and the rack force method, and the actual value is 0.02, with a relative error of 2.3%. Under the high-friction and large-steering-angle condition, the absolute error is 0.05, with a relative error of 5.9%. Under the low-friction and small-steering-angle condition, the absolute error is 0.017, with a relative error of 9.4%. Under the low-friction condition, the absolute error is 0.02, with a relative error of 6.25%. The proposed method can effectively estimate the road friction coefficient and achieve satisfactory results.

### 5.3. HIL Test Bench Comparison of the Dynamic Fusion Method and the Unscented Kalman Filter Algorithm Estimation Results

**Figure 14:**
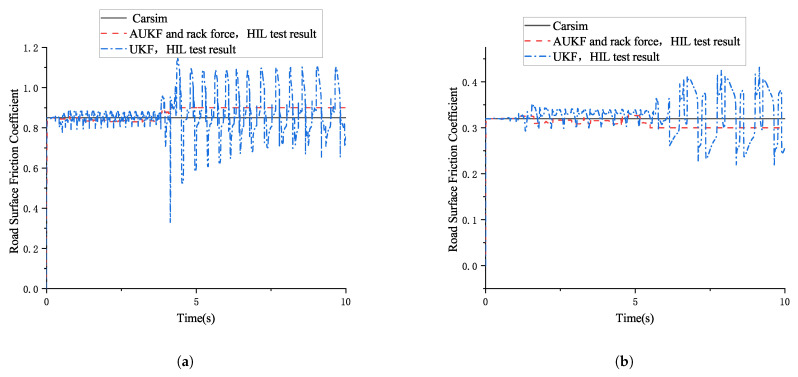
A comparison between the HIL test results of the road surface friction coefficient estimation based on the combination of AUKF and rack force and those of the traditional UKF algorithm. (**a**) HIL test result for road surface friction coefficient estimation (large front wheel angle high-friction coefficient). (**b**) HIL test result for road surface friction coefficient estimation (large front wheel angle low-friction coefficient).

Set the same operating conditions as in Section 5.2 in CarSim. A comparison between the HIL test results of the road surface friction coefficient estimation based on the combination of AUKF and rack force and those of the traditional UKF algorithm is shown in Figure 14.

The results of the steering-by-wire HIL test indicate that, across the entire steering angle range, whether under high- or low-friction conditions, the absolute and relative errors between the road surface friction coefficient estimated using the AUKF algorithm and rack force method and the road surface friction coefficient set in CarSim are smaller than those of the UKF algorithm. Additionally, across the entire steering angle range, the road surface friction coefficient estimated by the UKF algorithm fluctuates significantly due to the additional noise in the HIL test compared with the simulation. Clearly, the accuracy of the road surface friction coefficient estimation using the AUKF algorithm and rack force method is significantly higher than that of the UKF algorithm.

## 6. Conclusions

This paper combines the Adaptive Unscented Kalman Filter (AUKF), the Dugoff tire model, the vehicle three-degrees-of-freedom dynamic model, and the steer-by-wire actuator dynamics model to propose a method for estimating the road surface friction coefficient based on multimodal vehicle dynamics fusion. The method divides the front wheel angle into three intervals for segmental estimation, aiming to improve the estimation accuracy of the road surface friction coefficient within the large front wheel angle range.

For scenarios where the front wheel angle is less than 2.78°, the AUKF algorithm can accurately estimate the road surface friction coefficient, with a relative error of less than 1%. When the front wheel angle exceeds 3.2°, the rack force method provides a more accurate estimation of the road surface friction coefficient, with a relative error of less than 10%. For front wheel angles between 2.78° and 3.2°, the method combines the results from the AUKF algorithm and the rack force method, taking their average as the estimated road surface friction coefficient for this angle range, with a relative error of less than 7%. Comparative analysis shows that the segmental estimation method proposed in this paper outperforms the Unscented Kalman Filter (UKF) method in terms of estimation accuracy across different front wheel angle intervals.

The HIL test results of the steer-by-wire system validate the robustness and effectiveness of the algorithm proposed in this paper.

## Figures and Tables

**Table 1:** Steering system parameters.

Parameter	Value	Unit
Motor Moment of Inertia	0.00085	kg·m·m
Motor Viscous Damping Coefficient	0.00022	Nm·s/rad
Drive Shaft Torsional Stiffness	20,000	Nm/rad
Gearbox Reduction Ratio	16.5	-
Pitch Circle Radius	7.8	mm
Rack Mass	2.25	kg
Rack Damping	651	Nm·s/m

**Table 2:** Vehicle parameters.

Parameter	Value	Unit
Vehicle Mass	1765	kg
Distance from the Center of Gravity to Front Axle	1.2	m
Distance from the Center of Gravity to Rear Axle	1.4	m
Center of Gravity Height	0.5	m
Distance from Front Axle to Rear Axle	2.6	m
Track Width	1.6	m
Wheel Radius	0.354	m
Wheel Longitudinal Stiffness	10,803,737	N/rad
Wheel Lateral Stiffness	105,344	N/rad

**Table 3:** Analysis of road surface friction coefficient estimation results based on the combination of AUKF algorithm and rack force method.

Road Surface Friction Coefficient	Relative Error Between Simulation and Set Friction Coefficient in CarSim (Small Front Wheel Angle)	Relative Error Between Simulation and Set Friction Coefficient in CarSim (Small Front Wheel Angle)	Relative Error Between Simulation and Set Friction Coefficient in CarSim (Large Front Wheel Angle)	Absolute Error Between Simulation and Set Friction Coefficient in CarSim (Large Front Wheel Angle)
0.85	2.9%	0.025	5.9%	0.05
0.32	5.3%	0.017	6.25%	0.02

**Table 4:** Analysis of HIL test results for road surface friction coefficient estimation based on the combination of AUKF algorithm and rack force method.

Road Surface Friction Coefficient	Relative Error Between HIL Test Result and Set Friction Coefficient in CarSim (Small Front Wheel Angle)	Absolute Error Between HIL Test Result and Set Friction Coefficient in CarSim (Small Front Wheel Angle)	Relative Error Between HIL Test Result and Set Friction Coefficient in CarSim (Large Front Wheel Angle)	Absolute Error Between HIL Test Result and Set Friction Coefficient in CarSim (Large Front Wheel Angle)
0.85	2.3%	0.02	5.9%	0.05
0.32	9.4%	0.017	6.25%	0.02

## Data Availability

The data are contained within the article.
